# Complication Is Inevitable, but Suffering is Optional—Psychological Aspects of Dealing with Complications in Surgery

**DOI:** 10.1055/s-0043-1767830

**Published:** 2023-03-22

**Authors:** Hau D. Le, Justyna M. Wolinska, Reto M. Baertschiger, Sharifa A. Himidan

**Affiliations:** 1 Division of Pediatric Surgery, Department of Surgery, University of Wisconsin School of Medicine and Public Health, Madison, Wisconsin, United States; 2 Division of Pediatric Surgery, Department of Surgery, Children's Hospital of Eastern Ontario, University of Ottawa, Ottawa, Canada; 3 Division of General and Thoracic Surgery, Department of Surgery, University of Toronto, The Hospital for Sick Children, Toronto, Ontario, Canada; 4 Department of Surgery, Humber River Hospital, Toronto, Ontario, Canada

**Keywords:** complications, surgeons, psychological, well-being, second victim

## Abstract

Surgical complications remain common in health care and constitute a significant challenge for hospitals, surgeons, and patients. While they cause significant physical, financial, and psychological harm to patients and their families, they also heavily burden the involved physicians. This phenomenon, known as the “second victim,” results in negative short and long-term physical, cognitive, and psychological consequences on the surgeon. In this review, we explored the intricate connections between the surgeons' emotional response to adverse events concerning the patient outcome, perceived peer reaction, and existing social and institutional support systems. Using a selective literature review coupled with personal experiences, we propose a model of this complex interaction and suggest specific interventions to ameliorate the severity of response within this framework. The institution of the proposed interventions may improve the psychological well-being of surgeons facing complications and promote a cultural shift to better support physicians when they occur.

## Introduction

Surgical complications remain common in health care and constitute a significant challenge for hospitals, surgeons, and patients. Despite recent shifts in health care to better recognize system errors, implement safety checks, and ensure full disclosure, surgical complications continue to plague both patients and surgeons. Surgeons, whose training is long and arduous, are engaged health care workers who often take surgical complications as personal failures. The complex interaction between a surgeon's physical, emotional, and psychological response to adverse events concerning the patient's physical and psychological outcome, along with varying degrees of support from a surgeon's peers, institution, and supporting network, can result in deleterious psychological impacts. These very common psychological responses and the lack of support for physicians when they occur are known as the second victim syndrome. 1 This phenomenon can compromise technical skills and cognitive function, lead to anxiety or depression, and potentiate burnout which further damages well-being and future clinical practice. Here, we discuss the definitions of surgical complications, the psychological effects of complications on patients, and various models used to explain how complications affect surgeons, and we provide recommendations on minimizing their negative impact to improve the well-being of surgical specialists. Although this article is written for surgeons, it can be applied to health care providers from all specialties as the data are drawn from surgical and nonsurgical disciplines.

### Surgical Complication

*Definition and classification of surgical complication* : The term “surgical complication” is traditionally used to describe adverse and unexpected events that occur in surgery. However, there have been various attempts to redefine this term to better reflect the current health care environment and to be more applicable in all surgical practice types and specialties. Before 1992, there was no consistent definition of surgical complications. In 1992, Clavien et al recognized the vast differences in this definition and began to define it as “any deviation from the normal postoperative course.” 2 They also started classifying surgical complications into four grades based on the severity level. They differentiated them into three types of adverse outcomes after surgery: (1) complication, (2) failure to cure, and (3) sequela. Based on this work, Dindo et al revised the Clavien classification system, also called the T92 (short for Toronto 1992) system, and validated its reproducibility. This revised classification system divides seven complication levels into five grades. 3

In 2008, Clavien and Dindo together redefined the term “surgical complication” as “any deviation from the ideal postoperative course that is not inherent in the procedure and does not comprise a failure to cure.” 4 This aligns with Brennan et al's definition of adverse medical events as “unintended injuries caused by medical management rather than the disease process.” 5 Other notable classifications of surgical complications are the Memorial Sloan Kettering Cancer Center severity grading system, which also modified Clavien's original classification system into five grades of complications, and the Accordion Severity Grading. 6 7 Remembering that the “ideal postoperative course” is subjective to time, place, surgeon's ability, patient factors, and the availability of existing resources is important. Since all of these factors are subject to change, the “ideal postoperative course” is a dynamic process that is time- and place-dependent.

*Prevalence of complication* : With more than 200 million operations performed each year globally, the operating room is one of the highest-risk environments for patients. 8 9 In 1984, the Harvard Medical Practice Study included more than 30,000 randomly selected discharges from 51 randomly selected hospitals in New York State and reported that adverse events occurred in 3.7% of the hospitalizations. 10 Shockingly, 58% of the adverse events were attributable to errors, and 28% were due to negligence. Surgical complications accounted for more than half of the adverse events. Similarly, a study of surgical services at a Veterans Affairs Medical Center in Virginia included 44,603 consecutive major operations and found that 5.4% of patients suffered complications, of which nearly one-half were caused by errors, contributing to 7.5% of all deaths. 11 This report was followed by a study by Gawande et al in 1999, which reported that 66% of all adverse events from 15,000 nonpsychiatric discharges from hospitals in Utah and Colorado were surgical. Among these surgical adverse events, 54% were preventable, and 5.6% resulted in death. 12 In the same year, the Institute of Medicine published its first report on medical errors, “To Err Is Human,” intending to initiate change within the health care system to make it safer. The result is a concerted multifaceted effort to improve safety and avoidable adverse events. 13 One of the successful efforts is the Surgical Safety Checklist led by the World Health Organization and safety expert Atul Gawande, which led to a reduction in the rate of deaths associated with surgery from 1.5 to 0.8% and a reduction in inpatient complications from 11 to 7% after the introduction of the checklist. 14 Despite these reductions, tens of thousands of patients still experience harm each year.

### Effects of Complications on Patients

It is well known that surgery's adverse effects and complications can negatively impact not only the patient but also their families and friends, who are often referred to as the first victims. There is a large and growing body of literature and public awareness of the burden caused by these adverse events and their impact on patient's physical, economic, and, more recently, psychological and spiritual well-being. Despite significant advances in overall surgical care, pain management, and anesthesia, adverse outcomes in surgery persist. Some of these adverse events are still characterized as “expected,” “known,” or “acceptable” complications, despite efforts to eliminate or reduce their incidence and impact. These complications include bleeding, scarring, surgical site infection, pneumonia, urinary tract infection, and other hospital-acquired infections. In some cases, these complications can lead to mortality independent of the primary surgical disease.

*Physical effects* : Since surgery involves anatomic interventions, the impact of surgical complications on patients is most evident at the physical level. Much like the Clavien–Dindo classifications, the degree of physical impact can vary from minimal to the most severe outcome, which is death. The most widely accepted classification of surgical complications by Clavien and Dindo defines the lowest grade of complication (grade I) as “any deviation from the normal postoperative course without the need for pharmacological treatment, or surgical, endoscopic, and radiologist interventions.” 3 An example of this complication is superficial wound infection, which can be treated relatively quickly by opening the wound for drainage and packing without requiring any further procedures or antibiotics. These complications typically have little to no long-term physical effects on the patients. The more serious complications would require pharmacological treatments or surgical interventions. Examples include bleeding requiring transfusion or deep vein thrombosis requiring anticoagulation or an inferior vena cava filter placement. Life-threatening events may even require intensive care unit stay, procedures, or reoperation and can lead to multiple organ failure. Although some patients recover fully from these complications, many will have long-term disabilities or disfigurement after discharge. The highest grade of complication (grade V) is the death of a patient.

### Financial and Economic Effects of Complications on Patients

Surgical complications have garnered more attention with the increased focus on the health care economy and financial barriers to providing care. At least 20% of patients are estimated to suffer postoperative complications. 15 Complications significantly burden patients' financial well-being through direct out-of-pocket expenses and loss of productivity and income. With limited resources and priority-based budgets, hospital funding is primarily consumed by the cost of complications, which competes with funding for other essential patient care services. While it is difficult to calculate the out-of-hospital cost, it has also been demonstrated that the measured cost of hospital care increases dramatically and is aligned with the Clavien–Dindo classification when complications occur. 16 In a recent study looking at the effect of complications on hospital and some early out-of-hospital costs following colonic resection, the impact of complications significantly increased cost and correlated with the Clavien–Dindo severity grade I to IV. 17 Specifically, costs increased by 15.8, 36.8, 169.4, and 240.1%, per respective increase in complication grade severity, respectively.

*Psychosocial and spiritual effects on patients* : While the physical effects of surgical complications on patients can be diagnosed, quantified, and measured by physicians and organizations to track safety and progress, the diffuse impact of surgical complications on patients transcends the physical level. It is now widely accepted that not only does surgery induce trauma, but complications profoundly compound the psychological effects of this trauma. This can lead to significant long-lasting, potentially debilitating effects on the emotional well-being and quality of life of patients and their familial or social support system.

Surgical complications may incite psychological distress, cognitive impairment, social troubles, and spiritual changes in the patient. Patients who undergo surgeries often have existing stress factors and worries in their lives. In addition, they often develop acute posttraumatic stress symptoms in the postoperative period due to in-hospital stress. 18 In a landmark publication and the first published systematic review of the effect of surgical complications on patients' well-being, Pinto et al concluded that in addition to the impact on postoperative recovery, complications induce psychological distress, anxiety, and depression. 19

The severity of this heightened angst tends to correlate with the surgery's perceived seriousness. For example, patients undergoing major surgery, such as heart transplantation, often have a significantly higher level of depression and anxiety, and generally have worse psychological well-being compared with healthy subjects. 20 Patients who undergo permanent colostomies also experience significant adverse physical, psychological, social, and sexual health. 21 As their state of mind is already affected by their underlying disease and the traumatic experience of surgery, the patient's psychological well-being may deteriorate further when complications occur. This may be because complications cause an increase in anxiety and a lack of confidence in the individual's ability to thrive and perform as well as they did before surgery. 22 23

Recognition of these psychological effects on patients is vital in developing strategies to diagnose, treat, and follow patients to reduce the harmful impact on their well-being. The National Childhood Traumatic Stress Network is credited for creating a toolkit to help health care providers identify and treat traumatic medical stress in children and their families. 24 This intervention and other holistic and spiritual approaches could be adopted and modeled across a wide range of surgical specialties to address psychological aspects of surgical complications. 25

### Effects of Complications on Surgeons

To understand how surgical complications affect surgeons, we need to understand the factors contributing to the severity of the outcome and how they interact. Complications themselves occur via a complex process with many contributing factors. As the health care system becomes more complex and involves many different caregivers and treatments, the potential for errors grows. This change in health care delivery and the modernization of some aspects of quality of care have triggered system improvement rather than solely appointing responsibility to specific individuals. This has somewhat shifted the full burden of psychological impact away from individual health care providers toward the “system.” However, surgical complications, especially those occurring in the operating room, are still perceived as the surgeon's responsibility. It is more difficult to hold the “system” accountable for errors associated with the “hands” of the surgeon. This may be why surgical culture remains particularly harsh in the criticism of its peers when complications occur.

There is a common external perception of surgeons being cold and emotionally isolated technicians. In truth, surgeons pay attention to relationships, express empathy for their patients' and caregivers' distress, and share strong values related to the performance of meaningful work. 26 Moreover, the invasive and irreversible nature of surgical treatment and highly physical responsibility for the surgical outcome is intensely personal, and it makes surgeons particularly vulnerable to the harrowing impacts of adverse events. 26 27 Despite the changes in health care, surgeons are still considered the “captain of the ship” in the operating room, thus bearing the ultimate responsibility. Within the trenches of surgical training, several armored expectations of character are also instilled. These include confidence, accountability, decisiveness, perseverance, and even perfection. 26 28 Surgeons are expected to project these traits and navigate an often unsympathetic and competitive culture prone to blame, where revealing errors may damage their professional reputation. 28 29 The collateral damage of a patient's adverse events on the surgeon has been a growing focus of qualitative research aimed at untangling the complex physical, physiological, emotional, and behavioral consequences of complications. 30

*Phases of injury and recovery among second victims* : The term “second victim” was coined by Albert Wu and pertained to the lack of support for doctors when adverse events occur. 1 It is estimated that up to 43% of health care providers have been second victims. 31 32 A study by Scott et al reported that 30% of medical students, physicians, or nurses reported personal problems within the past 12 months. 33 Risk factors for becoming a second victim are broad. Some are related to aptitude, such as a complication due to a lapse in judgment and concentration or a lack of experience, knowledge, or skill. 30 Others include suffering burnout, being female, feeling demoralized, unrewarded, or having a lack of balance between one's professional and personal life. 34 35 36 Deficiencies in health care system resources may also pose a risk. 1

Feeling responsible for the adverse outcome, second victims often experience a myriad of emotional and physiological responses. The initial physical symptoms of the second victim phenomenon relate to an adrenaline-induced stress response. They include tachycardia, tachypnea, hypertension, muscle tension, unease, sweatiness, agitation, physical tension, shakiness, or clumsiness. 26 28 34 37 The biological response is often accompanied by feelings of failure and sadness for the patient and themselves. 28 Physical manifestations of this stress may continue to linger as extreme fatigue and sleep disturbance. 37 Additional psychosocial symptoms may include depression, poor concentration, anxiety, flashbacks, hyper-excitability, frustration, anger, self-doubt, loss of confidence, grief, and remorse. 37 Other responses include wanting to hide, feeling professionally unworthy, or questioning one' specialty choice. 27 28 34 Cognitive impairments such as difficulty concentrating, poor decision-making, and compromised dexterity may also occur. 26

The effects of complications on surgeons can persist for months after the initial event. These second victims tend to suffer repetitive distraction by re-living the events, searching for a cause, and being concerned about personal reputation, thus negatively impacting their well-being, family life, as well as their clinical or academic work. 28 34 38 Residents also describe being less communicative and withdrawn after adverse events, negatively impacting home interactions. 39 Many surgeons fear medicolegal repercussions and compromised reputations. 27 Although most second victims can function professionally, with only a few becoming incapacitated to work, many likely suffer in silence. 40 Psychological stress may be further compounded by emotional distress from possible medicolegal proceedings, peer review processes, root cause analysis investigations, or potential repercussions from their licensing board. 37 41

Surgeons often employ different coping mechanisms to adjust to life after adverse events. The typical initial therapeutic outlet is to discuss the complication with trusted family, friends, or colleagues. 28 40 When choosing counsel, surgeons most often seek out a senior or trusted colleague. 38 42 However, the discussions usually center around technical or clinical factors rather than emotional repercussions. 38 Some physicians benefit from the full disclosure discussion with the patient's family, and report a resulting sense of forgiveness. 34 However, approximately 16 to 25% of surgeons or health care providers deal with complications entirely alone. 38 40 One of the main reasons for not seeking help is the concern for reputational damage. 43 According to several qualitative studies, those who avoid discussing the adverse event are more prone to feel exhausted or crave isolation. 44 Unfortunately, alcohol and substance use also increased in up to 10% of surgeons involved in complications. 38 40 Other coping mechanisms included humor, exercise, hobbies, religion, or taking a leave of absence. 30 38 40 43 45 Not all coping mechanisms are repressive or destructive, however. Many surgeons have a deep interest in learning from an adverse event. 38 Seeking subsequent quality improvement and instilling a practice change to improve outcomes were therapeutic and constructive coping mechanisms exhibited by surgeons who managed stress well. 28 30

The long-term psychosocial impact of an adverse event varies among surgeons. Generally, it is unfavorable, only sometimes improves with time, and can produce cumulative effects. 28 Possible long-term behaviors include becoming more defensive, exercising extreme caution to avoid similar complications, changing the scope of practice, or even considering early retirement. 28 38 In Patel et al's study, 30.1% of surgeons felt that the ability to recover or handle the emotional impact of an adverse event improved with time and experience. 40 Occasionally, denial of the severity of the event was noted. 34 46 The time frame of the various phases of response to an adverse event also depended on the magnitude of the complication. 34

#### Impact of Complications on Surgical Performance

Surgery is a complex performance undertaken by individuals who study their craft meticulously and train arduously. A well-performed operation is often compared with a musical symphony, performed with impeccable technical ability, excellent acumen, and mental stability. Immediately following a complication, surgeons described physical responses to stress as having all the adrenaline-induced symptoms. 28 Stress has dispersed effects on the cognition responsible for memory, recall, and attention. 47 These symptoms also impact surgical technical performance by creating feelings of shakiness, clumsiness, reduced dexterity, and a tendency for mistakes. 48 As anxiety and irritation further build amidst stress, there is a high urgency to think, decide, and act, thus leading to haste, which can be detrimental to patient outcomes. 48 Perhaps more importantly, stress impairs other nontechnical skills, such as communication and decision-making. 49 This phenomenon is very similar to the performance of athletes whose mental stress is considered an independent risk factor for sports injuries and poor performance. 50 With the additive impact of stress in conjunction with the societal and institutional demands of their infallibility, surgeons are highly vulnerable. Unfortunately, the multitude of destructive psychological effects of adverse events on surgeons often targets their defining skills: technical ability, cognitive performance, and mental acumen.

*Surgical complications and burnout* : Burnout is a form of personal distress affecting physicians at an alarmingly more commonly than nonmedical personnel. Burnout is defined as a state of emotional exhaustion, depersonalization, and a decreased sense of personal accomplishment. 51 52 In 2022, a Medscape Lifestyle report found that the overall burnout rate was 47% among U.S. physicians. 53 Similarly, a systematic review in 2021 showed that the burnout rate among UK surgical specialties was also high, with 32% of consultants and 59% of surgical trainees affected. 54 The relationship between surgical complications and burnout is synergistic, as one can lead to the other and vice versa. Not surprisingly, burnout, among other factors such as having young children, compensation based on productivity, longer hours, a larger number of nights on call, and younger age, has been linked to suboptimal outcomes. 55 56 A Medscape Lifestyle survey in 2017 reported that the threat of malpractice and compassion fatigue (overexposure to death, violence, and other loss in patients) were two of the leading causes of burnout. 57

### Factors Impacting the Severity of the Response

Researchers have found many factors that influence the severity of the response to adverse events from physicians. Here, we group them into four categories that greatly affect the outcomes.

*The expectation gap* : Just as a complication can be considered the gap between the ideal outcome and the unfortunate reality, not meeting expectations plays a significant role in determining the severity of the second victim's response to complications. Complications in younger patients with better baseline health and those undergoing elective procedures rather than emergent elicit more extreme emotions among surgeons. 27 34 39 42 This is likely due to the expectation that young, healthy patients should have a more substantial capacity for healing and a lower chance of adverse outcomes. One can also rationalize that inducing a temporary or permanent injury on a very young and otherwise healthy patient that may pose life-long debilitation increases distress. Again, the expectation is a major influence when complications arise in higher levels of residency training, where operating responsibility increases. 39 Given that each surgeon has their own perceived “ideal outcome” for a particular operation, the magnitude of the complication and severity of the patient outcome can amplify the severity of the response to a complication. Therefore, patient death and permanent disability create the most intense emotional responses. 40 42 Similarly, preventable and unexpected complications are more impactful as the expectation gap is more significant. 43

Moreover, deaths occurring in the perioperative period or in younger patients can further deepen the intensity of negative emotions. 44 One interesting phenomenon involving the care of pediatric patients is that although pediatric surgeons tend to have lower rates of burnout compared with other specialties, a large proportion still feel emotionally exhausted and depersonalized. 58 59 They may also be at greater risk of poor emotional recovery after an adverse event, given the young age of their patients and deeper connections to patient families. 60 As a result, many pediatric surgeons have difficulty dealing with adverse outcomes. 61

Surgeons can experience more stress when the expectation gap related to outcomes is high from their patients, peers, or home institutions. One such example is when hospitals place unrealistic expectations on surgical outcomes, such as the push for “zero harm,” which is unattainable and can lead to the unintended consequence of causing more harm to the second victims.

*Relationship with the first victim* : The next factor is the strength of the connection between the second victim and the first victim. Deeper preoperative relationships with patients and their families can amplify the response to adverse events. 27 34 39 This response mirrors our natural tendency to be more distraught when accidents happen to loved ones rather than strangers. Among residents, the intensity of the perceived personal involvement in the patient's care increases their emotional response to complications. Specifically, more time spent on longer hospital rotations strengthens the feeling of ownership. 39 In addition, those who tend to personalize their experiences more are prone to suffer more negative impacts of complications. 34

Although surgeons who are less directly involved in the care of a patient (such as a colleague who briefly scrubs into a case) can also be affected by their adverse event, the degree of impact is amplified by the strength of the physical and emotional connections with the patient. 34 Perhaps, this explains why surgeons have the strongest reaction and feel most responsible when their technical skill is related to the adverse outcome, given the direct physical harm they cause to patients who place trust in their care. 44

*Level of experience* : Professional experience influences the response to surgical complications in various ways. As surgeons progress through their careers, years of experience may help with their resilience and coping potential. Several studies suggest that early career complications elicit stronger emotions, particularly if no major complications were experienced during residency. 27 29 42 43 However, surgeons who report no negative emotions following adverse events all have greater than 10 years of experience. 29 Experienced surgeons may have coping strategies to deconstruct adverse events more effectively. Although senior surgeons may generally benefit from more professional support, some studies suggest no difference in emotional reactions to errors according to seniority. 30 35 According to Patel et al, only a third of surgeons state that their ability to cope improved as they became more experienced. 40 Again, using sports as an analogy, experience and seniority do not always translate into a better response. As athletes get older and past their peak, their overall performance begins to decline despite gaining experience given the gradual deterioration in physical capacity. Similarly, more senior surgeons experience a more rapid decline in their surgical skills despite their growth in expertise. As a result, the intensity of reactions increases again closer to retirement. 29 This reaction may also result from the cumulative impacts of the psychological trauma caused by adverse events over time.

*Personality and personal factors* : Individual character and degree of personal resilience can influence the severity of the emotional impact of adverse events. 27 42 A personality trait such as extraversion can help surgeons reach out to others for support, which can benefit their psychological outcomes. How female and male surgeons respond to adverse events is also a difference. More often, male surgeons suppress public emotions after a patient's death, which prolongs the emotional response. 44 In contrast, female surgeons more often personalize their experience. 34 Women, minorities, and trainees usually hold fewer positions of power and are often marginalized, making them more vulnerable to impaired emotional recovery. 60 Those with a higher position in the hospital hierarchy may be less exposed to blame and, thus, be more protected from institutional investigation and root cause analysis. 42 46

#### Barriers to Seeking Support

Although the majority of surgeons eventually suffer the debilitating psychological consequences of complications, many endure this suffering without expressing it. The combined feelings of guilt, isolation, and failure amidst the surgical blame culture directly inhibit local disclosure, communication, and expression of emotions surrounding these events. 62 63 The traditional discussion of adverse events usually occurs during a morbidity and mortality (M&M) conference. The focus in these meetings centers around the technical components of the event that may result in accusation and rarely recognizes the surgeon as a possible victim. The consequence is an isolated, psychologically traumatized surgeon without social support. Hu et al describe that barriers to seeking support include lack of time (89%), uncertainty or difficulty with access (69%), concerns about lack of confidentiality (68%), negative impact on career (68%), and stigma (62%). 64

Although the degree, duration, and expression may vary, surgeon suffering after adverse events is unavoidable. There is a quiet but desperate cry for more of its recognition and even more urgency for cultural change to reduce its damage.

### Improving the Psychological Outcomes of Second Victims

Having a surgical complication is an inevitable part of a surgeon's career. There is an aphorism, “if you do not have a complication, it is because you have not operated long enough.”

To understand and optimize the well-being of surgeons in the aftermath of surgical complications, we need to understand the nature and dynamics of these psychological factors. The framework described in the above sections explains the psychological responses to surgical complications in both patients and surgeons and is based on the study of acute stress response and posttraumatic stress disorder (PTSD). Traditionally, PTSD was initially linked to the context of major traumatic events such as wars, terrorist attacks, victims of violence, and natural disasters. 65 However, PTSD can also occur following more common events such as medical procedures, bereavement, or employment-related stressors. 66 67 While PTSD is often considered an extreme psychological outcome when individuals are exposed to severe stress, other common results include depression and anxiety. A systematic review and meta-analysis in 2020 reported that the most prevalent symptoms manifested in health care providers following an adverse medical event were troubling memories (81%), anxiety/concern (76%), anger toward themselves (75%), regret/remorse (72%), distress (70%), fear of future errors (56%), embarrassment (52%), guilt (51%), and sleeping difficulties (35%). 68

With constant exposure to harmful and stressful experiences, one would expect that almost all surgeons would be dissatisfied with their careers. However, studies on the epidemiology of posttraumatic stress indicate that less than 10% of those exposed to traumatic stressors will develop full symptoms of PTSD. 69 Similarly, a survey by the American College of Surgeons in 2018 found that among 3,800 respondents, the career satisfaction rate among female and male surgeons was 77 and 82%, respectively. 70 Therefore, to improve the outcomes, we should focus on the positive aspects of this complex interaction and study the influential factors that explain why psychological distress varies between individuals after severe stressors.

In the next section, we propose a model where reactions to stress are generally influenced by three internal and three external factors. We suggest that individual surgeons have the potential to control the internal factors. The external factors, however, are influenced by everyone involved in or affected by the complication, some of which could be modifiable by a system or cultural changes. The basis of this model is developed from our own experience and literature review.

#### Internal Factors

*Understanding and experience* : The ability to clearly and objectively understand stressful life events is one of the key elements in the individual's response to them. To have a complete and clear understanding of the stressful event, one needs to understand the event itself and how the course of the event was affected by the perceived medical error. Let us take the example of a mucosal perforation during a laparoscopic pyloromyotomy that was recognized and repaired successfully. The ability to understand that this complication is a “known” and “acceptable” outcome for the surgeon is dependent on the following variables 1 : surgical judgment on the extent of the muscle being split, 2 condition of the pylorus (thick vs. thin), 3 type of surgery (laparoscopic vs. open), 4 and experience of the surgeon (novice vs. expert). After a complication occurs, a clear and objective understanding of how such complication might affect the patient and their families is essential in providing appropriate care and communication to the first victims. The correct appraisal of the situation is not necessarily the same for every surgeon but results from a combination of factors that include the surgeon's judgment, experience, and ability. Thus, the ability to understand the event without bias improves with experience, and the surgeon's experience plays a significant role in the intensity of the psychological impact of these events. Those with less experience should be well supported by trusted senior colleagues. Therefore, strong institutional efforts should be made to ensure that early career surgeons who lack experience are well-buffered and supported by the experience of senior surgeons.
*Situational awareness and concentration* : Surgeons possess a precious ability in their capacity to concentrate on the issue at hand. This proficiency enables them to perform effectively in high-stress and chaotic situations. They can see the order in the chaos because of their broad perspective of the entire situation (e.g., trauma resuscitation) while simultaneously lending focus to immediate acuity (e.g., performing an emergency thoracotomy while cardiopulmonary resuscitation is ongoing). This often requires surgeons to detach themselves emotionally from the situation. Interestingly, surgeons seem to lose this ability when complications occur. One possible cause is that complications can damage a surgeon's perceived identity. As reported by Biggs et al and Han et al, surgeons experience guilt, disappointment in themselves, and embarrassment when complications occur. These emotions prevent surgeons from detaching when necessary. Instead, they become part of the chaos, leading to suboptimal outcomes for the patients and themselves. To avoid the cycle of attachment and detachment, which creates emotional upheaval, one can strive for and cultivate equanimity: the state of psychological stability and evenness, undisturbed by extreme emotions and outside factors. The practice of mindfulness and meditation is one proven technique that can help surgeons achieve a state of equanimity while improving their capacity for situational awareness and concentration. 71 72 73 Surgeons with clear situational awareness also better know when to seek opinions from colleagues or transfer care to other surgeons if they cannot continue to provide unbiased or sufficient care.
*Ethical principles* : Surgeons choose which coping strategies they use based on the interaction between their understanding of the events and their awareness of the situation. A healthy coping mechanism will depend on sound ethical principles that should be upheld across all situations. For example, in response to a surgical complication, instead of following the “optimal” ethical principles of “taking care of oneself first, then others,” a surgeon may use alcohol or illicit substances to relieve distress caused by an adverse event temporarily. This might lead to additional problems, such as clouded judgment, impaired function, and future alcohol abuse, thus negatively affecting their well-being. Other negative ways surgeons might cope with the stress of surgical complications include denial, discounting, and emotional distancing. These are common coping strategies of less experienced staff after an adverse event. 74 These suboptimal responses can further reinforce societal mistrust in physicians, thus creating more tension between physicians and patients if complications occur. 75 Although many hospitals and institutions have established numerous bioethical faculties and ethics committees to help address this issue, there is also a danger that institutional ethical guidelines can be used to enforce policies against principled physicians who morally disagree with them. Physicians should be practitioners of modern ethical standards themselves. They should demonstrate that their actions and responses follow the principled ethical standards they have acquired through their own experience with mindfulness of other people's moral values. Finally, given that improving practice patterns or system deficiencies in light of a patient's complication is a well-recognized positive coping strategy, it should be encouraged and can similarly be guided by ethical standards.

#### External Factors

*Supporting network* : As the health care system becomes more complex, physicians are increasingly employed by large health care organizations. 76 Physicians within these complex health care systems have a decreased sense of autonomy and control over their work. 77 Many physicians feel they are just “cogs in the wheel” at their institutions. Nonetheless, the response from a surgeon's institutional supporting network is critical in preventing the negative impacts of complications attributed directly to their action. Lack of adequate institutional support after adverse events has detrimental impacts on physician well-being and is a major contributor to becoming a second victim. 1 78 79 Moreover, support from peers and supervisors is essential in alleviating negative psychological outcomes for surgeons after adverse events. 37 80 Similarly, emotional comfort from loved ones enhances a surgeon's well-being. Strategies aimed to support second victims include: having available peers to talk and listen, organizing structured and open discussion sessions of the complications, having senior or highly respected physicians to discuss their own complications and associated emotions, promoting compassion and empathy, providing a professional and confidential forum to discuss their complications besides M&M conferences and built-in checkups on colleagues who are recovering from complications. 1 80 81 82
Surgical culture has notoriously high expectations and is often shamelessly critical of others when errors occur. This culture perpetuates through generations of trainees who attempt to live up to its standard. As surgeon teachers, we educate trainees on the nature of complications and how to prevent them but fail to teach them how to support each other as colleagues when they occur. An opportunity to do this still lives within the heart of early surgical training, where emotional preparation for the inevitable world of complications could ignite a progressive movement in surgical culture toward intercollegial empathy.
We must be cognizant that any of us may become second victims or need to become supportive peers or supervisors. We must also amplify our sensitivity to recognize colleagues that may be prone to burnout or be more vulnerable to higher emotional stressors, such as trainees, early career surgeons, females, minorities, and anyone with lower positions of hierarchical power. Many of us are part of the institutions where second victims work. Many of us may be learning about the second victim phenomenon for the first time as seasoned practitioners. Most of us are comforted by knowing that we are not alone in our common emotional responses to adverse events. We must therefore continue to raise awareness of the second victim phenomenon, normalize its occurrence among health care professionals, and utilize these strategies to help remove the barriers to seeking support for the many physician victims around us.
*Patients' physical and psychological outcomes* : Outlined in the previous section are patient factors and physical outcomes that impact surgeons' response to complications based on differences in the expectation gap. Surgeons have some degree of control over their patients' psychological responses and outcomes after complications occur. First, they must be transparent and honest about the event. Hiding or minimizing their errors can lead to delayed treatment and more severe outcomes, while full disclosure can have therapeutic effects. Historically, medical education has placed most of its emphasis on medical knowledge and technical proficiency. Communication, though mandatory as a proficient skill, is not well taught. The unprepared and poorly delivered communication of bad news, including that of complications, further exacerbates their negative impact on patients, their families, and the surgeons. The recent push to standardize and train medical students in delivering bad news is an important initiative to improve the relationship between second and first victims, which can improve psychological outcomes for all those involved. Although it is more challenging to fully appreciate a patient's psychological state after an adverse event, their psychological outcomes tremendously impact a surgeon's well-being. Patients exhibiting peaceful acceptance of their outcome, even if suboptimal, can provide relief for surgeons, especially when a positive relationship between them persists.
*Society* : The disproportionate value society places on the physical and materialistic aspects of care creates an exaggerated response to surgical complications. In this standard, adverse surgical outcomes become focal points in the recovery of patients. Instead of a more holistic approach to relieving physical, mental, and spiritual suffering, we often prioritize and emphasize the physical aspect while neglecting the downstream mental and spiritual effects. For example, suppose we prioritize the cosmetic outcome of an operation above emotional well-being. Using this philosophy, any surgery that results in a perceived “ugliness” will induce negative emotions among patients, their loved ones, and the surgical team. Rethinking these priorities to include and prioritize emotional and spiritual well-being is needed to overcome the negative aspect of this type of complication. This does not mean that one should not care about the physical outcomes, as a disabled body resulting from a careless operation will cause emotional anguish for everyone involved. However, this change in priority will allow us to pay attention to the real drivers of our overall well-being.

## Conclusion

Recognition that complications are common, expensive, and significantly impact patients' physical and mental outcomes has resulted in a new era of health care reform emphasizing patient safety. Physicians and surgeons involved in adverse events or surgical complications often develop debilitating psychological effects that negatively impact their well-being, performance, and relationship with those around them. Using available data from medical and nonmedical fields combined with our own experience, we propose a model of interactions consisting of internal surgeon qualities alongside a series of external factors, both of which could be modified to reduce our suffering. Our strategies aim to improve each modifiable factor to counter the negative physiologic, cognitive, and emotional byproducts of complications. We anticipate that raising awareness of this issue will promote focused research and facilitate solutions where we lack evidence. The hope is to create meaningful and potentially universal interventions not only for surgeons but also for other health care providers. Among surgeons especially, a culture change aimed at normalizing the emotional response of complications and promoting an empathetic institutional support system is critical. With these proposals, we hope for a future where patients, surgeons, and other health care providers can ameliorate their suffering after adverse medical events and perhaps live by a paraphrased old Buddhist quote: “a complication is inevitable, but suffering is optional.”
